# Disruption in essential health services in Mexico during COVID-19: an interrupted time series analysis of health information system data

**DOI:** 10.1136/bmjgh-2021-006204

**Published:** 2021-09-01

**Authors:** Svetlana V Doubova, Hannah H Leslie, Margaret E Kruk, Ricardo Pérez-Cuevas, Catherine Arsenault

**Affiliations:** 1 Epidemiology and Health Services Research Unit CMN Siglo XXI, Mexican Institute of Social Security, Mexico City, Mexico; 2 Department of Global Health and Population, Harvard T. H. Chan School of Public Health, Boston, Massachusetts, USA; 3 Division of Prevention Science, University of California San Francisco, San Francisco, California, USA; 4 Division of Social Protection and Health, Inter-American Development Bank, Kingston, Jamaica

**Keywords:** health systems evaluation, maternal health, diabetes, hypertension, cancer

## Abstract

**Introduction:**

The COVID-19 pandemic has disrupted health systems around the world. The objectives of this study are to estimate the overall effect of the pandemic on essential health service use and outcomes in Mexico, describe observed and predicted trends in services over 24 months, and to estimate the number of visits lost through December 2020.

**Methods:**

We used health information system data for January 2019 to December 2020 from the Mexican Institute of Social Security (IMSS), which provides health services for more than half of Mexico’s population—65 million people. Our analysis includes nine indicators of service use and three outcome indicators for reproductive, maternal and child health and non-communicable disease services. We used an interrupted time series design and linear generalised estimating equation models to estimate the change in service use and outcomes from April to December 2020. Estimates were expressed using average marginal effects on the risk ratio scale.

**Results:**

The study found that across nine health services, an estimated 8.74 million patient visits were lost in Mexico. This included a decline of over two thirds for breast and cervical cancer screenings (79% and 68%, respectively), over half for sick child visits and female contraceptive services, approximately one-third for childhood vaccinations, diabetes, hypertension and antenatal care consultations, and a decline of 10% for deliveries performed at IMSS. In terms of patient outcomes, the proportion of patients with diabetes and hypertension with controlled conditions declined by 22% and 17%, respectively. Caesarean section rate did not change.

**Conclusion:**

Significant disruptions in health services show that the pandemic has strained the resilience of the Mexican health system and calls for urgent efforts to resume essential services and plan for catching up on missed preventive care even as the COVID-19 crisis continues in Mexico.

**Key questions**

**What is already known?**

- High-quality, resilient health systems should respond to crises while maintaining provision and quality of essential health services.
- In May to July 2020, the WHO and the Pan American Health Organization surveyed Ministries of Health on perceived disruptions in essential health services. Disruptions in health services were reported by nearly all countries.
- Little empirical evidence exists on the magnitude of the disruptions, particularly in low-income and middle-income countries.

**What are the new findings?**

- Using data from the health information system of the Mexican Institute of Social Security (IMSS), we found significant disruptions in reproductive, maternal and child health and non-communicable disease (NCD) services and outcomes during the first 9 months of the COVID-19 pandemic (April to December 2020).
- The most affected services included breast and cervical cancer screening (−79% and −68% declines), followed by sick child visits (−66%), contraceptive services (−54%), child vaccinations (−36%), diabetes and hypertension care (−32% declines in both) and antenatal care (−27%). Overall, we estimate that 8.74 million visits were missed from April to December 2020 across these nine health services, including approximately 1.1 and 0.5 million fewer women screened for cervical and breast cancer, respectively, 664 152 fewer antenatal care consultations and 4.2 and 2.0 million fewer consultations for diabetes and hypertension.

**Key questions**

**What do the new findings imply?**

- IMSS provides healthcare for more than half of Mexico’s population. The consequences of 8.74 million fewer patient visits for nine essential health services could be substantial if immediate measures are not taken to address these gaps.
- Our findings call for adoption of effective strategies to resume essential health services, adapt service provision during the pandemic (eg, telehealth) and catch up on missed healthcare including cancer screenings, child vaccinations and routine monitoring of patients with NCDs.

## Background

In a time of health crisis, high-quality, resilient health systems have two tasks: respond to the emergency and maintain provision and quality of essential health services.^1 2^ Reproductive, maternal and child health (RMCH) and non-communicable disease (NCD) care are essential health services that prevent avoidable morbidity and mortality.^1 3 4^

Since early 2020, the COVID-19 pandemic has strained governments and health systems, causing devastating economic and health consequences. Governments implemented heterogeneous strategies to limit the spread of COVID-19 and to withstand health shocks.^1 5 6^ Responses to COVID-19 included resource redistribution towards COVID-19, wide-scale testing, closures of non-essential businesses and schools, and physical distance restrictions (eg, curfews, lockdowns), among others.^5 7–9^

In May to July 2020, WHO and the Pan American Health Organization (PAHO) surveyed Ministries of Health on perceived disruptions in essential health services.^1 10^ Disruptions in health services were reported by nearly all countries, and more so in low-income and middle-income countries (LMICs) than high-income countries. The primary causes of reported disruptions were a mix of demand and supply factors, including health workforce-related disruptions (eg, clinical staff redeployment to provide COVID-19 relief or insufficient staff to provide services), fear of contagion and mistrust by the community, patients not presenting themselves at facilities and financial difficulties caused by the lockdowns.^1 10^ In addition, two studies from Nepal and Mexico on the early effects of the pandemic alerted of a potential upsurge in maternal deaths, stillbirths and neonatal deaths.^11 12^

Mexico is facing a heavy toll from the COVID-19 pandemic. In the year following the first known case on 28 February 2020, the disease had spread to almost 2.1 million people and killed more than 185 000 people, resulting in one of the highest case fatality rate in Latin America.^13^ COVID-19 cases increased steadily during 2020 in Mexico, with the majority of the new COVID-19 cases reported in the most populated areas, such as Mexico City and the State of Mexico followed by the states of Nuevo León, Guanajuato, Sonora and Jalisco.^14^ As a response to the pandemic, the Mexican government promoted physical distancing, but did not promote the use of face masks, nor did they perform massive-scale testing. Public and private health sectors repurposed multiple hospitals, reallocated health personnel and diverted medical equipment and supplies to treat COVID-19 patients. However, few strategies were implemented to ensure continuity of essential health services, including for RMNCH and NCD care.

The Mexican Institute of Social Security (IMSS) is the largest healthcare provider in Mexico. IMSS provides healthcare through its network of primary, secondary and tertiary care to more than half of the Mexican population—65 million people, primarily people employed in the formal labour market and their families. Whereas people without social security (such as informal workers or people living in remote areas) receive care at decentralised Ministry of Health facilities in each Mexican state or in the private sector. IMSS implemented a series of measures to respond to the pandemic. Since March 2020, 16 118 beds in 184 hospitals were repurposed to treat COVID-19 and 279 000 health workers were trained on COVID-19 care.^15^ IMSS also introduced a telephone line to provide COVID-19 information and implemented follow-up by phone for patients with suspected or confirmed COVID-19.

A study on the early indirect effects of the pandemic on maternal mortality in Mexico found an excess of 86 maternal deaths from all causes in the first 32 weeks of 2020, increasing the maternal mortality ratio to 42 per 100 000 live births from a predicted ratio of 29.5 per 100 000.^11^ Yet, there is currently no evidence on the indirect effects of the pandemic on health service provision in Mexico. To our knowledge, no studies have examined the effect of the pandemic on RMCH and NCD care service delivery in Mexico. Assessing the effect of the pandemic on essential health services is necessary to inform health system response. Evidence based on routinely collected data can accelerate local stakeholders and countries' learning on the effectiveness of health system adaptations to maintain essential health services.

The aims of this study are to estimate the overall effect of the COVID-19 pandemic on health service use and outcomes at IMSS, to describe observed and predicted trends in service use over 24 months, and to quantify the estimated number of visits lost due to the pandemic. Findings from this work can generate insights for addressing the indirect effects of the COVID-19 pandemic and inform current and future pandemic preparedness.

## Methods

We performed secondary data analysis of the IMSS health information system data.

### Data source

We used monthly data from the IMSS health information system for the period of January 2019 to December 2020 (24 months). The IMSS health system includes 1523 primary healthcare facilities and 283 hospitals located in 35 IMSS delegations across 32 Mexican states (one delegation per state except in Mexico City, the State of Mexico and Veracruz with two delegations per state). The IMSS health information system obtains information on a monthly basis for a range of performance indicators from all affiliated health facilities in the country. The information travels from health facilities to the Coordination of Information and Strategic Analysis in each delegation and finally to the headquarters of the IMSS Health Information Division in Mexico City. The capture and validation of the data are regulated by IMSS internal technical standards. The IMSS Health Information Division conducts regular, systematic validations of newly captured information and ensures plausibility of the data (information within allowed values and ranges) by communicating with delegations or facilities to verify discrepancies. Data for this study were available at the delegation level and there were no missing data.

### Measures

Our study includes 12 indicators on service use and outcomes covering nine RMCH and NCD services provided at IMSS (online supplemental table 1). Reproductive and maternal healthcare indicators included the number of reproductive-age women who used contraceptive services, number of antenatal care consultations, number of facility deliveries and the caesarean section (C-section) rate (calculated as the proportion of all deliveries performed by C-section). Child healthcare included the number of consultations for children under 5 with diarrhoea, pneumonia or malnutrition in primary care clinics, and the total number of children who completed the final required dose^16^ of the following vaccines: BCG vaccine; the rotavirus vaccine; the pentavalent vaccine against diphtheria, tetanus, pertussis, polio and Haemophilus influenzae type b; the pneumococcal vaccine and the measles, mumps and rubella (MMR) vaccine. Per the national vaccinationrecommendations,^16^ BCG should be administered at birth and the final dose of MMR should be delivered to 6 years old; the other vaccines should be administered during infancy. Vaccines were aggregated into one variable for the main analysis and analysed separately in online supplemental materials. Indicators for NCD services included the number of women screened for cervical (Papanicolaou test) and breast cancer (mammography), and the number of consultations for diabetes and hypertension among adults aged 20 or older at primary care clinics. Among patients with diabetes and hypertension, we also calculated the proportion with a controlled condition based on fasting blood glucose tests 70–130 mg/dL among patients with diabetes and on blood pressure tests <140/90 mm Hg among patients with hypertension. These 12 indicators reflect nine service utilisation indicators (absolute number of consultations) and three outcome indicators (C-section rate and proportion of patients with diabetes and hypertension with controlled blood sugar and blood pressure, respectively). These nine services represent the top reasons for consultations at IMSS^17^ and are aligned with the main causes of morbidity and mortality in Mexico.^18^

10.1136/bmjgh-2021-006204.supp1 Supplementary data

### Timeline

The first COVID-19 case was reported in Mexico on 28 February 2020, followed by the first COVID-19-related death on 19 March 2020. On 25 March 2020, the state of emergency was declared, and a series of containment policies were implemented at the national level including school closures and non-essential businesses closures. For this analysis, we define the pre-COVID-19 period as the months of January 2019 to March 2020 (15 months). The COVID-19 period includes April to December 2020 (9 months).

### Statistical analysis

Our final dataset included measures of service use and outcomes for each of the 24 months at the delegation level for all 35 IMSS Delegations. We used an interrupted time series design to estimate the average change in service delivery during the first 9 months of COVID-19.^19^ Each service indicator was regressed on an indicator variable for the COVID-19 period (where 0 is pre-COVID-19 and 1 is during COVID-19), time in months since January 2019 (to account for the overall trend), time in months since COVID-19 began (to account for the change in trend during COVID-19) and an indicator term for season to decrease potential confounding for seasonality (spring: March to May, summer: June to August, fall: September to November, winter: December to February). To account for the repeated observations over time, we used generalised estimating equation linear models with an identity link function and an exchangeable correlation structure. We used variograms to visually inspect the correlation structure for serial correlation and calculated the quasi likelihood under the independence model criterion (QIC) to identify the correlation structure with the smallest QIC (using the variog and qic commands in STATA V.16).^20 21^ Variograms did not show serial correlation. The exchangeable correlation structure had the smallest QIC and thus was chosen as the preferred correlation matrix in all models. To compare results across indicators with varying baseline, we estimated average marginal effects using the margins command in STATA and reported the level change as a risk ratio (RR). The analysis was performed at the delegation level and all models included robust standard errors. To visually assess indicators trends, we predicted average trends in the absence of the pandemic based on prepandemic observations and graphed them against observed data. Finally, we calculated the estimated number of visits lost during COVID-19 by comparing predicted volumes of services to observed volumes for each quarter (3-month segments) of the pandemic period (April to June 2020, July to September 2020 and October to December 2020). All analyses were conducted using STATA V.16.

### Patient and public involvement statement

Patients or the public were not involved in the design, or conduct, or reporting, or dissemination plans of our research.

## Results

The analytical dataset included 840 observations for the 35 delegations spanning 24 months. In total, these observations reflect 73.88 million patient visits for nine RMCH and NCD services provided from January 2019 to December 2020.

The provision of services at IMSS substantially decreased during COVID-19 (table 1). For example, before the pandemic, IMSS conducted an average of 77 436 first-time mammographies every month, compared with only 29 964 per month during COVID-19. NCD control among patients who visited IMSS facilities also declined during COVID-19. Before the pandemic, an average of 36% of patients with diabetes (aged 20+) had a controlled blood sugar during their visit at IMSS. During COVID-19, only 26% of patients with diabetes had a controlled condition at their consultation (table 1).

**Table 1:** Health service delivery pre-COVID-19 and during COVID-19, Mexican Institute of Social Security (IMSS), January 2019–December 2020

	Pre-COVID-19* N=15 months		During COVID-19 N=9 months	
	Average per month	SD	Average per month	SD
Reproductive and maternal healthcare*				
No of visits for contraceptives	43 270	7325	19 136	3217
No of antenatal care visits	285 007	17 494	196 455	10 039
No of facility deliveries (incl. caesarean sections)	32 634	3345	26 099	1366
Caesarean section rate (%)	45.18	0.86	46.86	0.84
**Child healthcare**				
No of consultations for sick children†	11 482	3321	2737	579
No of childhood vaccinations administered‡	97 729	11 615	49 307	11 130
**Chronic diseases care**				
No of women screened for cervical cancer§	216 808	15 826	84 752	26 005
No of women screened for breast cancer ¶	77 436	8933	29 964	15 106
No of consultations for diabetes care	1 335 635	63 419	926 302	126 467
Proportion of patients with diabetes with controlled condition (%)	35.68	1.22	26.38	2.35
No of consultations for hypertension care	1 414 592	126 182	1 016 122	175 488
Proportion of patients with hypertension with controlled condition (%)	67.83	6.80	54.76	8.71

*The pre-COVID-19 period is January 2019 to March 2020. The COVID-19 period includes April to December 2020.

†Includes consultations for diarrhoea, pneumonia and malnutrition in children under 5.

‡Number of children who received a unique dose of BCG vaccine, a third dose of pentavalent vaccine, a second dose of measles, mumps and rubella vaccine, a second dose of rotavirus vaccine and a third dose of the pneumococcal conjugate vaccine.

§Number of women aged 25–64 affiliated with IMSS screened with VIA for cervical cancer for the first time.

¶Number of women aged 50–69 affiliated with IMSS screened for breast cancer with a mammography for the first time.

**Figure 1:**
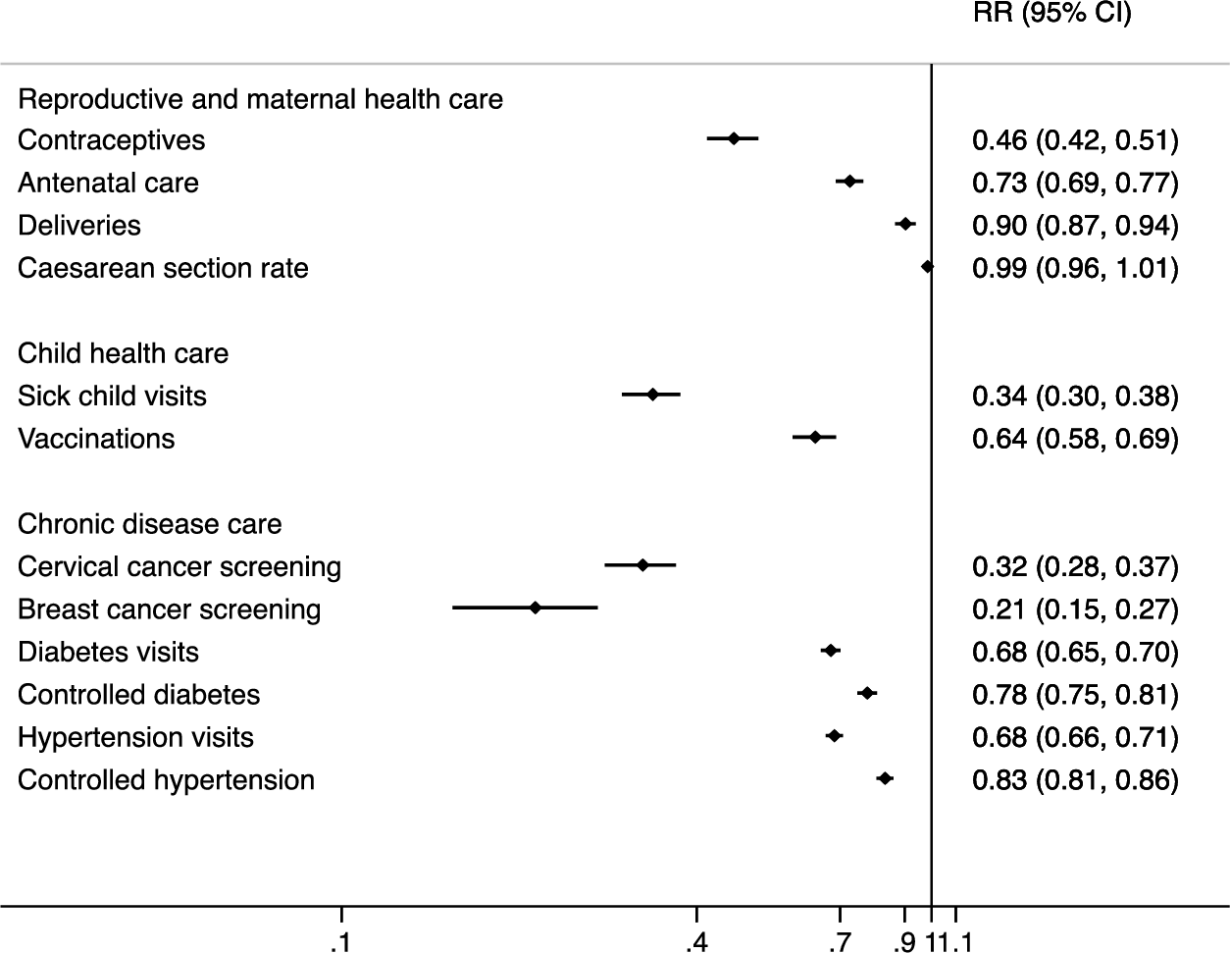
Risk ratios (RRs) for the average effect of COVID-19 (level change) on health service use and outcomes, Mexican Institute of Social Security, January 2019–December 2020. Risk ratios are plotted on the logarithmic scale. Detailed indicator definitions are in online supplemental material 1.

Figure 1 shows RRs from interrupted time series models for the level change in services during COVID-19. We found substantial declines in all indicators during COVID-19, ranging from −10% to −79%, with the exception of caesarean section rate.

In terms of service utilisation, breast and cervical cancer screening were the services most affected, declining by 79% and 68%, respectively, across delegations (RR 0.21, 95% CI 0.15 to0.27 and RR 0.32, 95% CI 0.28 to 0.37). Sick child visits and contraceptive services also declined by more than half during the first 9 months of the pandemic. Vaccinations declined by 36% (RR 0.64, 95% CI 0.58 to 0.69) while the number of diabetes, hypertension and antenatal care consultations declined by approximately one-third. The number of deliveries attended at IMSS declined by 10% (RR 0.90, 95% CI 0.87 to 0.94). We looked at the effect of the pandemic on each of the five childhood vaccines separately (online supplemental material). We found that four vaccines (BCG, pentavalent, rotavirus and pneumococcal) had similar declines ranging from 28% decline for rotavirus to 46% decline for pentavalent (RR 0.72, 95% CI 0.66 to 0.77 to RR 0.54, 95% CI 0.51 to 0.58). In contrast, there were no statistically significant decline for the number of children receiving the second dose of the MMR vaccine.

In terms of outcomes of care, the proportion of patients with diabetes and hypertension with controlled conditions declined by 22% and 17%, respectively (RR 0.78, 95% CI 0.75 to 0.81 and RR 0.83, 95% CI 0.81 to 0.86). C-section rates were not affected by the pandemic and remained stable at 45%–47%.

**Figure 2:**
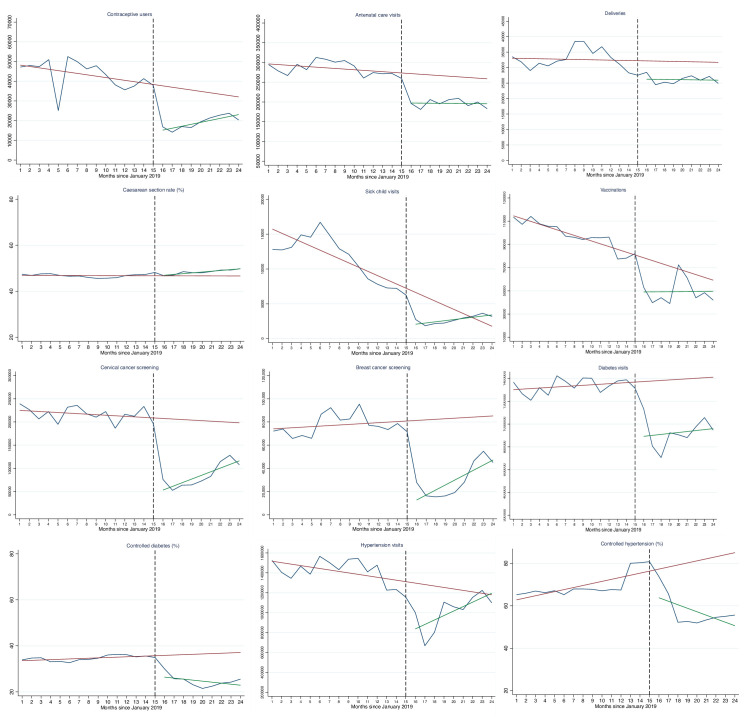
Trends in health service use and outcomes, Mexican Institute of Social Security, January 2019–December 2020. Red lines are the predicted linear trends from GEE models based on pre-COVID-19 months: January 2019–March 2020. Green lines are linear fitted lines for the 9 months during COVID. For a more intuitive visual interpretation the red and green lines do not account for seasonality. Blue lines are observed values. Black dotted line indicates the start of the COVID-19 period (starting April 2020). Months 1 to 24 correspond to January 2019 to December 2020. Indicator definitions are in online supplemental materials. Controlled diabetes and hypertension are among those who visited a primary care facility for a consultation only. GEE, generalised estimating equation.

Figure 2 shows observed and predicted trends over 24 months for all 12 indicators analysed. Several services such as vaccination and contraceptives services had negative trends before COVID-19 began (shown by the red line). Further change in service use and outcomes can be seen during the pandemic. By December 2020, 9 months into the pandemic, only 3 (sick child consultations, child vaccinations and consultations for hypertension) out of 12 indicators appear to have returned to pre-COVID-19 levels.

Comparing observed and predicted volumes of services suggests that an estimated 8.74 million patient visits were lost during the first 9 months of the pandemic across nine health services (table 2). This included an estimated 49 175 fewer deliveries attended, 488 153 fewer women screened for breast cancer, 1.1 million fewer women screened for cervical cancer and 4.2 and 2.0 million fewer consultations for diabetes and hypertension, respectively. For most services, the estimated number of visits lost was lowest in the last quarter of 2020 (October to December), indicating some resumption in service use by the end of 2020.

**Table 2:** Estimated number of patient visits lost during the COVID-19 pandemic (totals from April to December 2020) by quarter of 2020, Mexican Institute of Social Security

Indicator	2020 quarter	Observed	Predicted	Estimated difference	95% CI
Contraceptives services	2	47 999	107 948	59 949	57 426 to 62 471
	3	57 350	115 895	58 545	55 824 to 61 266
	4	66 871	98 483	31 612	29 263 to 33 960
Antenatal care visits	2	583 568	808 920	225 352	207 230 to 243 474
	3	610 712	840 821	230 109	211 477 to 248 741
	4	573 812	782 503	208 691	190 957 to 226 425
Deliveries	2	78 235	88 036	9801	7712 to 11 890
	3	78 673	98 728	20 055	17 587 to 22 523
	4	77 986	97 305	19 319	16 980 to 21 659
Sick child visits	2	6756	22 005	15 249	14 884 to 15 613
	3	7832	19 661	11 829	11 497 to 12 160
	4	10 045	7368	−2677*	−2 to 921 to −2 to 432
Vaccinations	2	136 807	230 260	93 453	88 237 to 98 670
	3	172 567	206 964	34 397	29 657 to 39 138
	4	134 388	186 608	52 220	47 436 to 57 004
Cervical cancer screening	2	192 674	602 223	409 549	392 342 to 426 756
	3	219 634	620 586	400 952	383 037 to 418 867
	4	350 456	590 371	239 915	219 317 to 260 512
Breast cancer screening	2	59 630	231 645	172 015	165 341 to 178 688
	3	63 664	269 346	205 682	197 873 to 213 491
	4	146 378	256 834	110 456	102 127 to 118 786
Diabetes visits	2	2 645 177	4 032 625	1 387 449	1 303 950 to 1 470 947
	3	2 711 435	4 237 637	1 526 202	1 438 732 to 1 613 671
	4	2 980 106	4 220 891	1 240 785	1 152 722 to 1 328 848
Hypertension visits	2	2 471 319	3 629 864	1 158 545	1 084 292 to 1 232 797
	3	3 197 036	3 851 904	654 868	575 742 to 733 994
	4	3 476 741	3 638 847	162 106	88 269 to 235 943

Quarter 2 is April to June 2020, quarter 3 is July to September 2020 and quarter 4 is October to December 2020. Total predicted is the sum of predicted visits from GEE models based on pre-COVID-19 months and adjusted for seasonality. Indicator definitions are in online supplemental materials.

*The negative estimated difference in sick child visits for quarter 4 of 2020 is due to observed visits in the fourth quarter surpassing predicted visits.

GEE, generalised estimating equation.

## Discussion

Using health information system data from the IMSS and an interrupted time series design, we found significant disruptions in essential health service use and outcomes during the COVID-19 pandemic. We estimated that across nine health services, a total of 8.74 million patient visits were lost in the first 9 months of the pandemic. Declines in service provision ranged from −79% for breast cancer screening to −10% for deliveries performed at IMSS.

At the beginning of the pandemic, IMSS recommended limiting health facility visits to emergencies such as severe respiratory symptoms. Most routine healthcare appointments were cancelled or postponed. IMSS also introduced a plan to reduce traffic in health facilities and to prevent COVID-19 transmission among high-risk groups including pregnant women and people with chronic illnesses.^22^ The plan involved paid maternity leave for pregnant women (with an online application process), refillable electronic prescriptions for patients with chronic disease and a telephone line for COVID-19 information and medical advice.

IMSS also repurposed a large number of hospital beds to COVID-19 care. In an effort to maintain maternal health services, IMSS signed a contract with private hospitals to conduct 28 000 deliveries and C-sections for IMSS affiliates.^23^ However, no alternatives for other RMNCH services including antenatal care were introduced (eg, telemedicine). Our findings indicated that an estimated 49 175 fewer deliveries took place in IMSS facilities, nearly twice the number covered by the private sector contract. This indicates that the rest of deliveries not performed by IMSS were most likely paid out-of-pocket by the women themselves in private sector facilities, although data from the private sector are not available to confirm this.

Consistent with studies from several other countries,^24^ there were no significant changes in the C-section rate at IMSS during the first 9 months of the COVID-19 pandemic. The C-section rate at IMSS has been historically high: 45% on average in 2019, which is substantially higher than the OECD countries average of 28%.^25^ This figure is consistent with the rate at the national level in Mexico.^26 27^ Previous studies identified that in Mexico, both physicians and women perceive C-section as a safer and more convenient mode of delivery compared with vaginal birth.^26–28^ Thus, C-sections are frequently performed at the woman’s request.^28^ There was a possibility that C-sections might increase during COVID-19 as providers and women would prefer a more rapid mode of delivery due to fear of contagion in health facilities. In addition, C-sections might be preferred for women with COVID-19 to mitigate the potential risk that excessive ventilation and stress during labour might aggravate the respiratory and pro-inflammatory status accompanying the disease.^29^ In China, an increase in C-section rate was observed among COVID-19 infected women.^29^ Our findings suggest no net changes in C-section rate at IMSS.

Prior to the pandemic, several RMCH services were underperforming in Mexico. For instance, use of modern contraceptives was only 65.5%^30^ in 2015, and immunisation coverage among children under 5 ranged from 56% for the third dose of hepatitis B vaccine to 86% for the third dose of pneumococcal conjugate vaccine in 2018.^31^ Low vaccination rates prior to the pandemic were partially caused by vaccine shortages observed since 2014.^32^ Declining trends in vaccination and contraceptive services before the pandemic began were also found in the present study. These declining trends are alarming and mirror the overall underperformance of the national vaccination plan of the public healthcare sector in Mexico.^33^ Access to vaccines and contraceptives is intended to be universal in Mexico and not tied to social security affiliations. However, we found disruptions in the provision of these services that surpassed the predicted trends.

The observed 54% decline in contraceptives is worrisome and might increase the rate of unplanned pregnancies. Reductions in family planning services were reported in other LMICs including declines ranging from 10% to 74% in Burkina Faso, Kenya, Nigeria and Pakistan.^34^ In contrast, another recent study found an increase in contraceptive use among women in need in rural Burkina Faso and rural Kenya.^35^

Declines in antenatal care coverage, child vaccination and sick child care may place the IMSS population at risk for excess maternal and child morbidity and mortality not attributable to COVID-19. An increase in maternal mortality has already been reported in Mexico.^11^ The consequences of missed RMCH services need further evaluation. A recent modelling study of the potential indirect effects of COVID-19 found that a 6-month reduction in service coverage of the magnitude seen in this study could result in a 10%–45% increase in under 5 deaths per month and in an 8%–39% increase in maternal deaths per month.^36^ In the last decade, Mexico had reached near universal coverage for antenatal care (98.6%) and institutional deliveries (94.5%).^37^ The maternal mortality ratio was 33 per 100 000 live births in 2017, and the under-5 mortality rate was 14.2 per 1000 live births in 2019,^38 39^ surpassing the 2030 Sustainable Development Goals targets for these indicators. Failing to address the gaps in service coverage caused by the pandemic could set back these hard-earned gains.

There is also a particular concern about the mid-term to long-term effects of disruptions in routine immunisation that put children at risk for vaccine-preventable diseases.^40 41^ We estimate that 180 070 fewer childhood vaccinations were provided at IMSS among the five vaccines analysed for the period of April to December 2020. The decline in vaccination was observed for BCG, pentavalent, rotavirus and pneumococcal vaccines in newborns and infants, and is particularly worrying given prepandemic declines in vaccination among this age group. It is possible that newborns delivered in private facilities may have received BCG outside of IMSS. Following-up with caregivers of infants to confirm vaccination status should be considered as a priority for IMSS providers. Measures should be taken immediately to immunise unvaccinated children to avoid larger outbreaks of these vaccine-preventable diseases. This will require adequate vaccine supply and interventions to promote routine visits and ensure complete vaccination.

Chronic diseases are highly prevalent in Mexico, where more than 70% of adults are overweight, and an estimated 12% and 45% have diabetes and hypertension, respectively.^42^ Diabetes, cardiovascular diseases and cancer are the top causes of ambulatory and hospital care, disability and premature mortality in the country.^42 43^ Prior to COVID-19, unmet need for NCD care was high and effective coverage (defined as quality-adjusted coverage) for patients with NCD was estimated to be lower than 50%.^44^

Patients with NCDs require continuous and timely care to prevent complications and improve their quality of life. To maintain NCD care during the pandemic, IMSS offered electronic 3-month refillable prescriptions for patients with NCD with controlled conditions, assuming that their routine consultations could be delayed. Yet, no options for regular monitoring were proposed for those with uncontrolled conditions (eg, patients with high blood glucose or high blood pressure). The present study found that during the first 9 months of the pandemic, routine consultations for diabetes and hypertension declined by 32%.

The decline in primary care visits for people with diabetes and hypertension in our study coincided with a significant decline in controlled fasting glucose and controlled blood pressure among IMSS patients. It is worth noting that these indicators are calculated among patients who visited facilities. We found that prior to the pandemic, only 36% of IMSS patients with diabetes and 68% of patients with hypertension had a controlled condition, which is similar to previous national estimates of NCD control for diabetes and better for hypertension^45 46^

The declines in NCD control observed at IMSS since the pandemic could be the result of either worsening disease among IMSS patients (caused by a lack of medical care and follow-up) or that visits were limited to those with more severe conditions, while those with controlled diseases used refillable prescriptions without attending primary care clinics. We believe that both mechanisms are likely at play. IMSS should take action to verify the disease control status for all users in routine care. Blood pressure and glycosylated haemoglobin, or fasting glucose should be tested for those renewing prescriptions virtually. For example, a biannual visit may be required for all users.

Our findings for NCD care disruptions during the pandemic concur with the experience of other countries.^10 34^ For instance, in 28 Latin American and Caribbean countries, PAHO found that 11% of countries interrupted, and 64% limited, access to NCD outpatient services.^10^ The analysis of essential health services in Burkina Faso, Ethiopia, India, Kenya, Nigeria and Pakistan revealed 10% to 49% drop in NCD services in 2020, compared with 2019 and 2018.^34^ A survey from Spain also revealed^7^ that 67% of chronic disease patients had their consultations and laboratory tests postponed or cancelled during the first wave of the COVID-19 pandemic (April–June 2020). In addition, 10% of respondents reported postponing their consultations or tests themselves due to the fear of contagion while 44% indicated that their health had worsened.^7^

The decline in patients with controlled diabetes and hypertension at IMSS could result in excess morbidity, avoidable hospitalisations and mortality from both COVID-19 infection and chronic disease complications. For instance, multiple studies showed that uncontrolled diabetes and hypertension are important predictors of COVID-19 severity and mortality.^47–49^ In patients with poor glycaemic control, each 1 mmol/L of fasting plasma glucose can increase the risk of COVID-19 severity by 33%^48^; while hypertension can increase the risk by 27%.^49^ In addition, glycaemic control in patients with diabetes, and blood pressure control in patients with hypertension, are the primary mechanisms for preventing acute and chronic complications and consequent disability and premature mortality.^50 51^ Therefore, the decline in NCD service provision and outcomes found in this study could have serious and long-lasting effects in Mexico given the size of the population that IMSS covers. Excess hospitalisations and deaths in patients with chronic disease must be urgently monitored and addressed during and after the COVID-19 pandemic.

Breast and cervical cancer screening were also substantially affected, and we found no evidence of ‘catching-up’ for these missed screenings. Declines in breast and cervical cancer screening could lead to diagnosis and treatment delays and may increase the proportion of avoidable deaths. Other countries are facing similar declines in cancer screening. A national population-based modelling study from England has projected that the COVID-19 pandemic may lead to an 8%–10% increase in 5-year breast cancer deaths, a 15%–17% increase in colorectal cancer-related deaths, and a 5% increase in lung cancer deaths due to delays in cancer diagnoses.^52^ Important declines in cervical and breast cancer screening were also reported in the USA.^53 54^

Our study has strengths and limitations. We conducted a comprehensive assessment of health service trends during the pandemic in Mexico and provided a framework for other countries to analyse health system performance during COVID-19. The 12 indicators analysed covered a wide range of health needs for people at different ages and relate to the most important causes of morbidity and mortality in Mexico.^17 18^ Therefore, findings from this study may be of considerable interest to health systems researchers and policy-makers around the world who face similar challenges during the pandemic.

Even though, our study includes nine essential health services indicators that represent the top reasons for consultations at IMSS, other services such as mental healthcare, dialysis for patients with chronic kidney disease or services for patients with HIV, may have been further affected by the pandemic and were not included. Second, the interrupted time series design controls for pre-COVID-19 trends but does not account for events other than the pandemic, which may have affected these trends (eg, change in government policies, funding or vaccine shortages). Third, the data used in this study are from the IMSS health information system, an administrative data source that may contain errors. Nonetheless, the IMSS health information system staff conduct regular follow-up with health facilities and systematic validation of indicators. The pandemic may have affected reporting quality of the data. However, the health information division did not report changes in data completeness and quality. Fourth, our study included 15 months before COVID-19 and 9 months during the pandemic. Regular monitoring of health system performance should continue to ensure that health services resume. Finally, we were unable to assess the extent to which IMSS affiliates turned to the private sector for care during the pandemic. Such assessment can be relevant, as the IMSS population is generally better off than the population covered by the Ministry of Health facilities and may be more likely to seek services in private facilities.

## Conclusions

We found that IMSS—the health system covering half of Mexico’s population—experienced a significant failure of resilience during the COVID-19 pandemic. Large disruptions in RMCH and NCD services were observed at IMSS during the first 9 months of the pandemic and most services have not returned to pre-COVID levels. To reduce avoidable morbidity and mortality, IMSS must resume the provision of essential health services as soon as possible despite the ongoing COVID-19 crisis affecting the country. WHO^55^ released a guide with recommendations for modifying service delivery and adapt essential health services to the changing context of the pandemic while accelerating resumption in health services. Recommended strategies include adjustments to governance and coordination mechanisms to provide essential services; strengthening communication strategies to support appropriate use of essential services by the population; use of digital platforms to increase access to care and provide effective care at a distance through telehealth (eg, online, phone calls or short message service). Telehealth strategies have been found to be effective in providing patient counselling and education, screenings, follow-up and refilling prescriptions, and have led to significant improvements in health outcomes.^56–58^ However, the majority of studies on the effectiveness of telehealth were performed in high-income settings. There is an urgent need to assess the acceptability and effectiveness of telehealth for care in LMIC contexts. IMSS and stakeholders in Mexico must identify and implement effective and culturally accepted strategies to quickly resume essential health services. In January–March 2021, WHO conducted a second round of the pulse survey on continuity of essential health services during the COVID-19 pandemic.^59^ Overall, 94% of countries surveyed continued to report some kind of disruption but the magnitude and extent of disruptions had decreased. However, countries in the WHO Region of the Americas reported the highest average percentage of services disrupted.^59^ Much work remains to improve pandemic preparedness in the region and improve the resilience of the IMSS and similar health systems.

## Data Availability

Data are available in a public, open access repository. The study database is publicly available from the Harvard Dataverse Repository at: https://dataverse.harvard.edu/dataset.xhtml?persistentId=doi:10.7910/DVN/XSHQYB. The statistical code is available at: https://github.com/catherine-arsenault/Disruptions-IMSS-COVID.

## References

[1] World health Organization. Pulse survey on continuity of essential health services during the COVID-19 pandemic interim report. WHO reference number: WHO/2019-nCoV/EHS_continuity/survey/. Geneva: World health Organization, 2020.

[2] Kruk ME, Myers M, Varpilah ST, et al. What is a resilient health system? Lessons from Ebola. Lancet 2015;385:1910–2. 10.1016/S0140-6736(15)60755-3 25987159

[3] Alam N, Mamun M, Reproductive DP. Child, and Adolescent Health (RMNCAH): Key Global Public Health Agenda in SDG Era. In: Leal Filho W, Wall T, Azul A, eds. Good health and well-being. encyclopedia of the UN sustainable development goals. Cham: Springer, 2019.

[4] World Health Organization. Trends in maternal mortality: 2000 to 2017: estimates by WHO, UNICEF, UNFPA, world bank group and the United nations population division. Geneva: World Health Organization, 2019. https://www.who.int/reproductivehealth/publications/maternal-mortality-2000-2017/en/

[5] Chi Y, Regan L, Nemzoff C. Beyond COVID-19: a whole of health look at impacts during the pandemic response. CGD policy paper 177. Washington, DC: Center for Global Development, 2020. https://www.cgdev.org/publication/beyond-covid-19-whole-health-lookimpacts-during-pandemic-response

[6] World Health Organization,. COVID-19 strategic preparedness and response plan (SPRP) operational planning guidelines to support country preparedness and response. Geneva: World Health Organization, 2020.

[7] Galvez M, Rueda Y, Gomariz V. Estudio del impacto de covid-19 en las personas con enfermedades crónicas: Informe de resultados. [Study of the impact of covid-19 in people with chronic diseases: Report of results. Madrid: Plataforma de Organizaciones de Pacientes, 2020. https://www.plataformadepacientes.org/estudio-del-impacto-de-covid-19-en-las-personas-con-enfermedad-cronica

[8] Ziedan E, Simon KI, Wing C. Effects of state COVID-19 closure policy on NON-COVID-19 health care utilization. NBER working paper No. 27621. National Bureau of Economic Research Inc, 2020. https://www.nber.org/system/files/working_papers/w27621/w27621.pdf

[9] Malhotra C, Chaudhry I, Ozdemir S. Reduced health-care utilization among people with chronic medical conditions during coronavirus disease 2019. Proc Singapore Health 2021:2010105820964533. 10.1177/2010105820964533

[10] Pan American Health Organization. Rapid assessment of service delivery for NCDS during the COVID-19 pandemic in the Americas. Washington: Pan American Health Organization, 2020. https://iris.paho.org/handle/10665.2/52250

[11] Lumbreras-Marquez MI, Campos-Zamora M, Seifert SM, et al. Excess maternal deaths associated with coronavirus disease 2019 (COVID-19) in Mexico. Obstet Gynecol 2020;136:1114–6. 10.1097/AOG.0000000000004140 32909969

[12] Kc A, Gurung R, Kinney MV, et al. Effect of the COVID-19 pandemic response on intrapartum care, stillbirth, and neonatal mortality outcomes in Nepal: a prospective observational study. Lancet Glob Health 2020;8:e1273–81. 10.1016/S2214-109X(20)30345-4 32791117 PMC7417164

[13] Johns Hopkins University of Medicine. Coronavirus resource center. mortality in the most affected countries. Baltimore: Johns Hopkins University of Medicine, 2020. https://coronavirus.jhu.edu/data/mortality

[14] Statista. Número de casos confirmados de coronavirus (COVID-19) en México al 22 de diciembre de 2020, POR entidad federativa. México: estados más afectados por el coronavirus 2020. Statista Research Department, 2021. https://es.statista.com/estadisticas/1109201/numero-casos-coronavirus-mexico-estado/

[15] Government of Mexico. IMSS, fundamental institution and axis of Mexico’s response to the COVID-19 pandemic. No. 786/2020. Government of Mexico [Internet]. Available: http://www.imss.gob.mx/prensa/archivo/202011/786 [Accessed 23 Nov 2020].

[16] Ministry of Health. Vaccination manual. Mexico: Ministry of Health, 2017. [Manual de vacunación. México: Secretaría de Salud. Centro Nacional para la Salud de la Infancia y la Adolescencia, 2017], 2017. Available: https://www.gob.mx/salud%7Ccensia/documentos/manual-de-vacunacion-edicion-2017

[17] Government of Mexico. IMSS. Report to the federal executive and the Congress of the Union on the financial situation and risks of the Mexican Institute of social security 2019-2020. Mexico: Instituto Mexicano del Seguro Social, 2020. http://www.imss.gob.mx/conoce-al-imss/informe-2019-2020

[18] Institute for Health Metrics and Evaluation. Global Burden of Disease Study 2016. Viz Hub. Disability-adjusted life years per 100 000. Both sexes. [Internet]. Available: https://vizhub.healthdata.org/gbd-compare

[19] Wagner AK, Soumerai SB, Zhang F, et al. Segmented regression analysis of interrupted time series studies in medication use research. J Clin Pharm Ther 2002;27:299–309. 10.1046/j.1365-2710.2002.00430.x 12174032

[20] Cui J. QIC program and model selection in Gee analyses. Stata J 2007;7:209–20. 10.1177/1536867X0700700205

[21] Diggle P, Heagerty P, Liang K-Y. Analysis of longitudinal data. 2nd edn. UK: Oxford, 2002.

[22] Government of Mexico. IMSS presents a plan to prevent COVID-19 infections in pregnant women, the elderly and patients with chronic diseases. No. 163/2020. Government of Mexico [Internet]. Available: http://www.imss.gob.mx/prensa/archivo/202003/163 [Accessed 1 Apr 2020].

[23] Government of Mexico. With the subrogation agreement to private hospitals, up to 28 thousand deliveries and caesarean sections of pregnant women entitled to the IMSS will be attended.No.223/2020. Government of Mexico [Internet]. Available: http://www.imss.gob.mx/prensa/archivo/202004/223 [Accessed 26 Apr 2020].

[24] Chmielewska B, Barratt I, Townsend R, et al. Effects of the COVID-19 pandemic on maternal and perinatal outcomes: a systematic review and meta-analysis. Lancet Glob Health 2021;9:e759–72. 10.1016/S2214-109X(21)00079-6 33811827 PMC8012052

[25] OECD. Health at a Glance 2019: OECD Indicators. Caesarean sections, OECDiLibrary. [Internet], 2020. Available: https://www.oecd-ilibrary.org/sites/fa1f7281-en/index.html?itemId=/content/component/fa1f7281-en

[26] Suárez-López L, Campero L, De la Vara-Salazar E. Características sociodemográficas y reproductivas asociadas con el aumento de cesáreas en México [Sociodemographic and reproductive characteristics associated with the increase of cesarean section practice in Mexico]. Salud Publica Mex 2013;55:S225–34. 24626699

[27] Guendelman S, Gemmill A, Thornton D, et al. Prevalence, disparities, and determinants of primary cesarean births among first-time mothers in Mexico. Health Aff 2017;36:714–22. 10.1377/hlthaff.2016.1084 28373338

[28] Bernal-García C, Nahín-Escobedo Campos C. Cesarean section: current situation and associated factors in Mexico. Revista Salud Quintana Roo 2018;11:28–33.

[29] Vouga M, Grobman WA, Baud D. More on clinical characteristics of pregnant women with Covid-19 in Wuhan, China. N Engl J Med 2020;383:696–7. 10.1056/NEJMc2016881 32790269

[30] Ponce de Leon RG, Ewerling F, Serruya SJ, et al. Contraceptive use in Latin America and the Caribbean with a focus on long-acting reversible contraceptives: prevalence and inequalities in 23 countries. Lancet Glob Health 2019;7:e227–35. 10.1016/S2214-109X(18)30481-9 30683240 PMC6367565

[31] World Health Organization. WHO vaccine-preventable diseases: monitoring system. 2020 global summary. Mexico: World Health Organization [Internet], 2020. https://apps.who.int/immunization_monitoring/globalsummary/countries?countrycriteria%5Bcountry%5D%5B%5D=MEX&commit=OK

[32] Mongua-Rodríguez N, Hubert C, Ferreira-Guerrero E. Tendencias en las coberturas de vacunación en niños de 12 a 23 y 24 a 35 meses en México [Trends in vaccination coverage among children aged 12-23 and 24-35 months in Mexico. Ensanut 2012 and Ensanut 100]. Salud Publica Mex 2019;61:809–20. 31869545 10.21149/10559

[33] Hernández-Ávila M, Palacio-Mejía LS, Hernández-Ávila JE. Vacunación en México: coberturas imprecisas y deficiencia en el seguimiento de los niños que no completan el esquema [Vaccination in Mexico: imprecise coverages and deficiency in the follow-up of children with incomplete immunization]. Salud Publica Mex 2020;62:215–24. 32237565 10.21149/10682

[34] PATH. Essential health services during and after COVID-19: a sprint analysis of disruptions and responses across six countries. PATH, 2020. Available: https://path.azureedge.net/media/documents/EHS_sprint_intro_and_full_report_revised-compressed.pdf [Accessed 20 Jul 2021].

[35] Wood SN, Karp C, OlaOlorun F, et al. Need for and use of contraception by women before and during COVID-19 in four sub-Saharan African geographies: results from population-based national or regional cohort surveys. Lancet Glob Health 2021;9:e793–801. 10.1016/S2214-109X(21)00105-4 34019835 PMC8149322

[36] Roberton T, Carter ED, Chou VB, et al. Early estimates of the indirect effects of the COVID-19 pandemic on maternal and child mortality in low-income and middle-income countries: a modelling study. Lancet Glob Health 2020;8:e901–8. 10.1016/S2214-109X(20)30229-1 32405459 PMC7217645

[37] González-Pier E, Barraza-Lloréns M, Beyeler N, et al. Mexico's path towards the Sustainable Development Goal for health: an assessment of the feasibility of reducing premature mortality by 40% by 2030. Lancet Glob Health 2016;4:e714–25. 10.1016/S2214-109X(16)30181-4 27596038 PMC5024342

[38] World Health Organization. Maternal mortality in 2000-2017. Mexico. internationally comparable MMR estimates by the maternal mortality estimation Inter-Agency group (MMEIG) who, UNICEF, UNFPA, world bank group and the United nations population division. Geneva: World Health Organization, 2019. https://www.who.int/gho/maternal_health/countries/mex.pdf?ua=1

[39] The World Bank data. Mortality rate, under-5 (per 1,000 live births) – Mexico. Available: https://data.worldbank.org/indicator/SH.DYN.MORT?locations=MX

[40] World Health Organization, American Red Cross U.S, CDC, UNICEF, UN Foundation. More than 117 million children at risk of missing out on measles vaccines, as COVID-19 surges. Geneva: World Health Organization, 2020.

[41] O'Leary ST, Trefren L, Roth H, et al. Number of childhood and adolescent vaccinations administered before and after the COVID-19 outbreak in Colorado. JAMA Pediatr 2021;175:305–7. 10.1001/jamapediatrics.2020.4733 33284331 PMC7921904

[42] National Institute of Public Health. Midway National health and nutrition survey 2016. (ENSANUT 2016) final report of results. Mexico: National Institute of Public Health, 2016.

[43] The Global Cancer Observatory. Cancer today. [homepage on the internet] Publishing WHOweb. [Cited 2020 April 26]. Available: https://gco.iarc.fr

[44] Leslie HH, Doubova SV, Pérez-Cuevas R. Assessing health system performance: effective coverage at the Mexican Institute of social security. Health Policy Plan 2019;34:ii67–76. 10.1093/heapol/czz105 31723962

[45] Basto-Abreu A, Barrientos-Gutiérrez T, Rojas-Martínez R. Prevalencia de diabetes y descontrol glucémico en México: resultados de la Ensanut 2016 [Prevalence of diabetes and poor glycemic control in Mexico: results from Ensanut 2016. Salud Publica Mex 2020;62:50–9. 31869561 10.21149/10752

[46] Campos-Nonato I, Hernández-Barrera L, Flores-Coria A, et al. [Prevalence, diagnosis and control of hypertension in Mexican adults with vulnerable condition. Results of the Ensanut 100k]. Salud Publica Mex 2019;61:888–97. 10.21149/10574 31869552

[47] Williamson EJ, Walker AJ, Bhaskaran K, et al. Factors associated with COVID-19-related death using OpenSAFELY. Nature 2020;584:430–6. 10.1038/s41586-020-2521-4 32640463 PMC7611074

[48] Lazarus G, Audrey J, Wangsaputra VK, et al. High admission blood glucose independently predicts poor prognosis in COVID-19 patients: a systematic review and dose-response meta-analysis. Diabetes Res Clin Pract 2021;171:108561. 10.1016/j.diabres.2020.108561 33310127 PMC7725108

[49] Zhang J, Wu J, Sun X, et al. Association of hypertension with the severity and fatality of SARS-CoV-2 infection: a meta-analysis. Epidemiol Infect 2020;148:e106. 10.1017/S095026882000117X 32460927 PMC7270484

[50] Ratner RE. Glycemic control in the prevention of diabetic complications. Clin Cornerstone 2001;4:24–37. 10.1016/S1098-3597(01)90027-4 11838325

[51] Lewington S, Clarke R, Qizilbash N, et al. Age-Specific relevance of usual blood pressure to vascular mortality: a meta-analysis of individual data for one million adults in 61 prospective studies. Lancet 2002;360:1903–13. 10.1016/s0140-6736(02)11911-8 12493255

[52] Maringe C, Spicer J, Morris M, et al. The impact of the COVID-19 pandemic on cancer deaths due to delays in diagnosis in England, UK: a national, population-based, modelling study. Lancet Oncol 2020;21:1023–34. 10.1016/S1470-2045(20)30388-0 32702310 PMC7417808

[53] Miller MJ, Xu L, Qin J, et al. Impact of COVID-19 on Cervical Cancer Screening Rates Among Women Aged 21-65 Years in a Large Integrated Health Care System - Southern California, January 1-September 30, 2019, and January 1-September 30, 2020. MMWR Morb Mortal Wkly Rep 2021;70:109–13. 10.15585/mmwr.mm7004a1 33507893 PMC7842810

[54] Kuehn BM. Dramatic cervical cancer screening decline during pandemic. JAMA 2021;325:925. 10.1001/jama.2021.1977 33687473

[55] World Health Organization. Maintaining essential health services: operational guidance for the COVID-19 context: interim guidance, 1 June 2020. Geneva: World Health Organization, 2020. Available: https://apps.who.int/iris/handle/10665/332240

[56] Murugesu S, Galazis N, Jones BP, et al. Evaluating the use of telemedicine in gynaecological practice: a systematic review. BMJ Open 2020;10:e039457. 10.1136/bmjopen-2020-039457 PMC7722813 33293306

[57] Omboni S, McManus RJ, Bosworth HB, et al. Evidence and recommendations on the use of telemedicine for the management of arterial hypertension: an international expert position paper. Hypertension 2020;76:1368–83. 10.1161/HYPERTENSIONAHA.120.15873 32921195

[58] De Groot J, Wu D, Flynn D, et al. Efficacy of telemedicine on glycaemic control in patients with type 2 diabetes: a meta-analysis. World J Diabetes 2021;12:170–97. 10.4239/wjd.v12.i2.170 33594336 PMC7839169

[59] World Health Organization. Second round of the National pulse survey on continuity of essential health services during the COVID-19 pandemic: January-March 2021. Interim report. Geneva: World Health Organization, 2021.

