# Supplementary material for: Disruption in essential health services in Mexico during COVID-19: an interrupted time series analysis of health information system data
Source: BMJ Glob Health. 2021 Sep 1;6(9):e006204. doi: 10.1136/bmjgh-2021-006204 (PMC8413469; doi:10.1136/bmjgh-2021-006204)
Supplement: Supplementary data [file bmjgh-2021-006204supp001.pdf]

Disruption in essential health services in Mexico during COVID-19: an interrupted time series analysis

Supplementary materials

Supplementary Table 1. Detailed definitions: service delivery indicators extracted from the Mexican Institute for Social Security (IMSS) routine health information system

Indicator	Numerator	Denominator
**Reproductive and maternal health care**		
Contraceptive users	Number of reproductive-age women who used contraceptive services and accepted a contraceptive prescription for the first time or for a refill	-
Antenatal care visits	Total number of antenatal care visits (including first and subsequent visits)	-
Total facility deliveries	Number of facility deliveries + Number of caesarean sections	-
C-section rate %	Number of caesarean sections	Number of facility deliveries + Number of caesarean sections
**Child health care**		
Number of consultations for sick children	Number of under-5 children visited primary care clinics for sick childcare including for diarrhea, pneumonia or malnutrition	-
Number of childhood vaccinations administered	**BCG vaccine** (Number of children vaccinated with a unique dose of BCG)	-
	**Pentavalent vaccine** (Number of children vaccinated with a 3rd dose pentavalent + Number of children vaccinated with a 3rd dose of pentavalent acellular)	-
	**MMR vaccine** (Number of children vaccinated with the second dose of the triple viral measles, mumps and rubella vaccine)	-
	**Pneumococcal vaccine** (Number of children who received the third dose of pneumococcal vaccine)	-
	**Rotavirus vaccine** (Number of children who received the second dose of the rotavirus vaccine)	-
**Chronic disease care**		
Screened for cervical cancer	Number of women aged 25 - 64 screened with Papanicolaou test for cervical cancer	-
Screened for breast cancer	Number of women aged 50 - 69 screened for breast cancer for the first time with a mammography	-
Diabetes visits	Number of diabetic patients visited primary care clinics (20+ years)	-
Controlled diabetes %	Number of diabetic patients (20+years) with fasting blood glucose tests 70–130 mg/dl	Number of diabetic patients visited primary care clinics (20+years)
Hypertension visits	Number of hypertensive patients visited primary care clinics (20+ years)	-
Controlled hypertension %	Number of hypertensive patients (20+years) with blood pressure <140/90mmHg	Number of hypertensive patients visited primary care clinics (20+years)

Doubova SV, *et al. BMJ Global Health* 2022; 6:e006204. doi: 10.1136/bmjgh-2021-006204

Supplementary Table 2. Childhood vaccines administered pre- and during COVID-19, Mexican Institute of Social Security, January 2019-December 2020

	Pre-COVID-19 [a] N= 15 months		During COVID-19 N= 9 months	
**Childhood vaccines**	**Average per month**	**SD**	**Average per month**	**SD**
BCG vaccine	21,800	2,420	9,881	9,347
Third dose of the Pentavalent vaccine	48,608	8,902	17,823	1,594
Second dose of the MMR vaccine	6,436	4,302	6,468	2,313
Second dose of the Rotavirus vaccine	16,196	1,108	12,024	1,769
Third dose of the Pneumococcal vaccine	4,688	444	3,111	745

[a] The Pre-COVID-19 period is January 2019 to March 2020. The COVID-19 period includes April to December 2020
BCG is Bacillus-Calmette Guérin
MMR is Measles, Mumps and Rubella

Doubova SV, *et al. BMJ Global Health* 2022; 6:e006204. doi: 10.1136/bmjgh-2021-006204

Supplementary figure 1. Risk ratios for the average effect of COVID-19 (immediate level change) on childhood vaccinations, Mexican Institute of Social Security, January 2019-December 2020

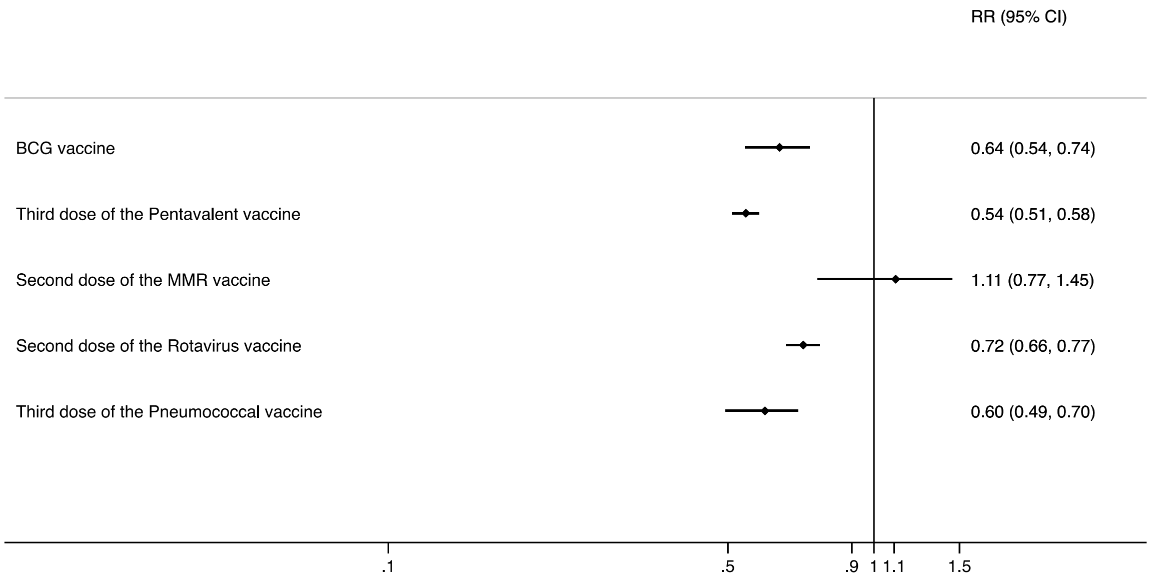

BCG is Bacillus-Calmette Guérin
MMR is Measles, Mumps and Rubella
